# Chemical dampening of Ly6C^hi^ monocytes in the periphery produces anti-depressant effects in mice

**DOI:** 10.1038/srep19406

**Published:** 2016-01-19

**Authors:** Xiao Zheng, Sijing Ma, An Kang, Mengqiu Wu, Lin Wang, Qiong Wang, Guangji Wang, Haiping Hao

**Affiliations:** 1 State Key Laboratory of Natural Medicines, China Pharmaceutical University, Nanjing 210009, Jiangsu, China; 2 Department of Pharmacy, Nanjing University of Chinese Medicine Affiliated Hospital, Nanjing 210029, Jiangsu, China; 3 Department of Pharmacy, Affiliated Hospital of Hunan Academy of Chinese Medical Sciences, Changsha 410000, Hunan, China; 4 School of Pharmacy, Nanjing University of Chinese Medicine, Nanjing 210023, Jiangsu, China

## Abstract

The involvement of systemic immunity in depression pathogenesis promises a periphery-targeting paradigm in novel anti-depressant discovery. However, relatively little is known about druggable targets in the periphery for mental and behavioral control. Here we report that targeting Ly6C^hi^ monocytes in blood can serve as a strategy for anti-depressant purpose. A natural compound, ginsenoside Rg1 (Rg1), was firstly validated as a periphery-restricted chemical probe. Rg1 selectively suppressed Ly6C^hi^ monocytes recruitment to the inflamed mice brain. The proinflammatory potential of Ly6C^hi^ monocytes to activate astrocytes was abrogated by Rg1, which led to a blunted feedback release of CCL2 to recruit the peripheral monocytes. *In vitro* study demonstrated that Rg1 pretreatment on activated THP-1 monocytes retarded their ability to trigger CCL2 secretion from co-cultured U251 MG astrocytes. CCL2-triggered p38/MAPK and PI3K/Akt activation were involved in the action of Rg1. Importantly, in mice models, we found that dampening Ly6C^hi^ monocytes at the periphery ameliorated depression-like behavior induced by neuroinflammation or chronic social defeat stress. Together, our work unravels that blood Ly6C^hi^ monocytes may serve as the target to enable remote intervention on the depressed brain, and identifies Rg1 as a lead compound for designing drugs targeting peripheral CCL2 signals.

Depression is a highly prevalent yet poorly treated mental disorder. The pathogenesis involves complicated gene-environment-psyche interactions, and immune dysfunction has emerged as a key component in this pathological network^1^. In comparison to the neurotransmitter-centered view of depression, an important conceptual advance of the immune theory of depression is the appreciation of a deregulated immune network at the systems-level^2^,^3^,^4^. Indeed, experimental and clinical studies have shown that psychiatric symptoms are largely accompanied by changes in immune cells and molecules both in the brain and periphery^5^,^6^,^7^. In particular, peripheral immune mediators could engage in a bidirectional interaction with brain-resident cells^3^,^8^,^9^, which exerts a fine control over the behavioral and mental outcome. These emerging findings suggest that brain-targeted therapeutics may not suffice to alleviate the depression syndromes as a systems disorder. Given the intricate role of the peripheral immune system in shaping mood and behavior, as well as the inherent limitation of brain-targeted interventions, an attractive therapeutic approach is to harness the immune-to-brain signaling at the periphery^4^. Actually, although still in the infancy, a few, yet limited, successful cases have been reported that promise a periphery-targeting strategy to treat central nervous system (CNS) diseases such as neurodegeneration and cognitive impairment^10^,^11^.

To validate the peripheral-targeting paradigm to combat psychiatric disorders such as depression, an important task is to define druggable targets in the peripheral immune system. However, to enable an efficient and safe control, there are multiple challenges given the phenotypical and functional heterogeneity of the systemic immune mediators. For example, it remains controversial whether the blood-borne cells such as T cells and monocyte subsets recruited to the brain exacerbate brain injuries or, conversely, confer protective and repairing benefits^12^,^13^,^14^,^15^. Also, the translational landscape of peripheral targets to effective therapeutics against central pathologies remains to be depicted. To fill these knowledge gaps, chemical probes that could specifically target key immune cell subsets in the periphery are highly needed.

Natural products have historically served as excellent sources of biologically active compounds and continue to provide novel templates in the search for new drugs^16^,^17^. Ginseng is such a natural product with a long historical use in the treatment of brain disorders. However, a long-standing paradox is that ginsenosides, the major active components, are largely characterized with poor brain transport property and very low distribution to the CNS. We have previously shown that anti-inflammatory mechanism in the periphery explained the neuroprotective effect of total ginsenoside extract^18^. Recently, we further elucidated that peripheral immunomodulatory effects may explain the central benefits of ginsenoside Rg1 (Rg1), one of the major ginsenoside components^19^. We therefore reason that Rg1may serve as a brain non-penetrating chemical probe to identify potential targets for combating brain disorder from the periphery.

In this work, by validating and employing Rg1 as a peripherally-restricted chemical probe, we identified Ly6C^hi^ monocytes as a peripheral target to protect against depressive behavior. In a mice model of neuroinflammation-associated depression, we discovered that Rg1 worked as a specific inhibitor against the brain recruitment of Ly6C^hi^ monocytes. Mechanistic investigation revealed that Rg1 disrupted the downstream signaling of CCL2-CCR2 interaction and dampened the proinflammatory potential of Ly6C^hi^ monocytes. Importantly, in depressive mice induced by chronic social defeat stress, we further evidenced that dampening Ly6C^hi^ monocytes in blood was sufficient to ameliorate depression and anxiety-like behaviors.

## Results

### Peripherally-restricted Rg1 alleviates neuroinflammation-associated behavioral impairment

**Figure 1:**
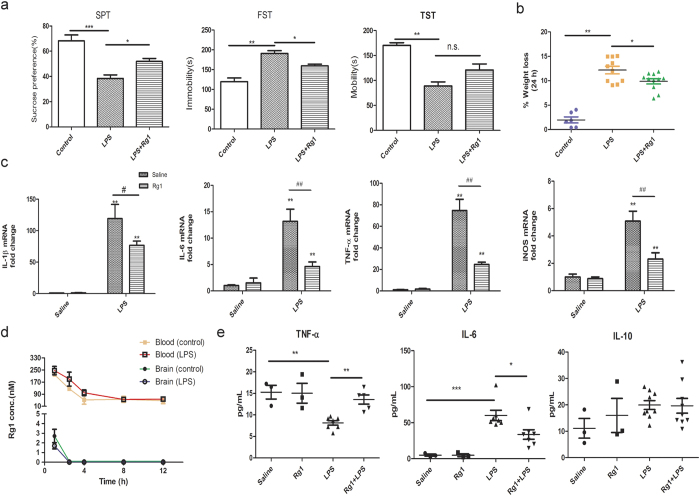
Rg1 ameliorates neuroinflammation and associated behavioral deficits in mice. Mice received vehicle or Rg1 (20 mg/kg, i.p., once daily for 4 consecutive days) before an intracerebroventricle (i.c.v.) lipopolysaccharide (LPS, 3 μg) challenge. At 24 h later, the mice were evaluated for the depression-like behaviors. (**a**) Behavioral assessment of mice 24 h after the LPS challenge in the forced swimming test (FST), sucrose-preference test (SPT) and tail-suspension test (TST). n.s., not significant. (n = 6–8 mice/group). (**b**) The weight loss of mice 24 h after central LPS injection. (**c**) Quantitative PCR analysis of proinflammatory mediators (IL-1β, IL-6, TNF-α, iNOS) in the mice brain. ^*^P < 0.05, ^**^P < 0.01, compared to corresponding saline control group; ^#^*P* < 0.05, ^##^*P* < 0.01, LPS/saline group compared to LPS/Rg1 group. (**d**) Rg1 concentration dynamics determined in the blood and brain of LPS-challenged mice (1, 2.5, 4, 8, 12 h). (**e**) Blood levels of typical cytokines (TNF-α, IL-6, IL-10) in mice 24 h after central LPS challenge (n = 4–6 mice/group). Data represent mean ± s.e.m. ^*^P < 0.05, ^**^P < 0.01, ^***^P < 0.001.

To verify the validity of Rg1 for probing anti-depressive targets at the periphery, the pharmacological effects and distribution profile of Rg1 in a mice model with neuroinflammation-induced depression-like behavior were investigated. In line with previous reports^20^,^21^, LPS-challenged mice showed a pronounced anhedonia and depression-like behavior (Fig. 1a) as well as weight loss (Fig. 1b). In contrast, mice receiving Rg1 (20 mg/kg, i.p.) displayed a significant improvement of behavioral deficits in the sucrose preference test and forced swimming test (*P* < 0.05). Also, a trend for increased mobility time was observed for Rg1-treated mice in the tail suspension test, although this did not reach statistical significance compared with LPS-challenged mice. In addition, Rg1 treatment effectively attenuated the metabolic disturbance of serotonin and kynurenines, which are endogenous metabolites closely involved in behavioral regulation (Supplementary Fig. S1a). Consistent with the anti-depressive benefits, PCR analysis of typical proinflammatory mediators (IL-6, IL-1β, TNF-α, iNOS) showed a marked suppression of neuroinflammatory disturbance by Rg1 (Fig. 1c). In line with the anti-inflammatory effects, Rg1 treatment led to a significant improvement in neuronal survival (Supplementary Fig. S1b).

Importantly, we observed that Rg1 was concentrated in the periphery but hardly detectable in the LPS-inflamed brain, only exhibiting a peak cerebral level around 1.7 ± 0.3 nM (Fig. 1d and Supplementary Fig. S1c). According to previous reports, such a concentration is far below the minimum level of Rg1 (>10 μM) required to confer a direct anti-inflammatory or neuroprotective effect^22^,^23^. Of interest, we also noted that neuroinflammation-induced disturbance of typical cytokines in blood was effectively attenuated in mice receiving Rg1 (Fig. 1e), which was in line with the peripheral action of Rg1. These findings verify that Rg1 could effectively ameliorate neuroinflammatory disturbance and depression-like behavior without penetrating into the brain. Therefore, Rg1 could serve as an appropriate chemical probe to identify potential peripheral targets for neuroinflammatory and behavioral intervention.

### Rg1 selectively mitigates Ly6C^hi^ monocyte infiltration to the brain

**Figure 2:**
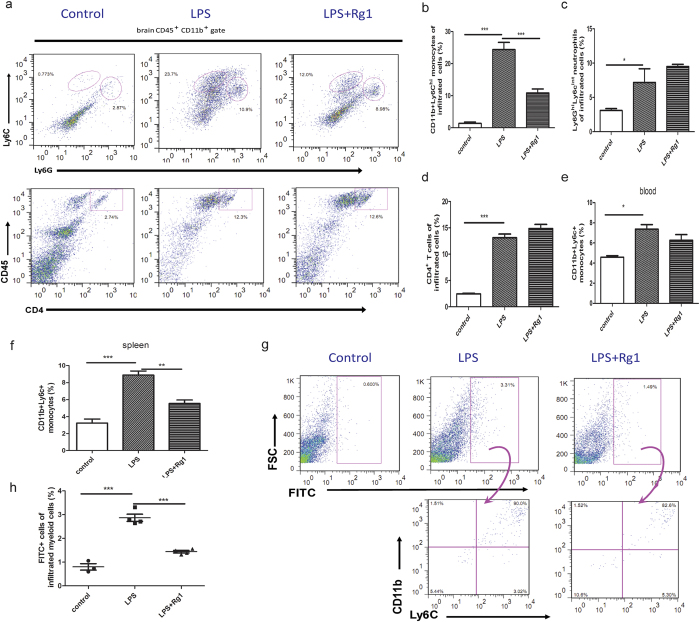
Selective abrogation of the brain accumulation of Ly6C^hi^ monocytes by Rg1. Mice received vehicle or Rg1 (20 mg/kg, i.p.) before central LPS challenge (3 μg). The brain, spleen and blood were harvested 24 h later and processed for flow cytometric analysis. (**a**) Quantitative analysis of brain infiltrated CD4^+^ T cells gated by CD45^hi^CD4^+^. (**b**) Quantitative analysis of brain infiltrated neutrophils gated by CD45^hi^CD11b^+^Ly6G^hi^Ly6C^int^. (**c**) Quantitative analysis of brain infiltrated Ly6C^hi^ monocytes gated by CD45^hi^CD11b^+^Ly6C^hi^. (**d)** Quantitative analysis of Ly6C^hi^ monocyte frequency in mice blood. (**e**) Quantitative analysis of Ly6C^hi^ monocyte frequency in mice spleen. (**f**) Flow cytometric analysis of FITC-labeled Ly6C^hi^ monocytes in the inflamed brain. The Ly6C^hi^ monocytes from the periphery were identified by CD11b^+^FITC^+^. (**g**) Comparison of the frequency of brain-infiltrated FITC^+^Ly6C^hi^ monocytes. (n = 4–6 mice/group). Data represent mean ± s.e.m. ^*^P < 0.05, ^**^P < 0.01, ^***^P < 0.001.

To identify the peripheral “target” that enables the indirect effect of Rg1 on the brain, we focused on the peripheral blood where Rg1 maintained a high level and long retention. Specifically, we asked whether Rg1 may influence the behavior of circulating immune cells, in light of the fact that they are key sources of cytokines and versatile mediators in the brain-periphery crosstalk machinery. Flow cytometric analysis revealed significantly increased proportions of immune cell infiltrates in LPS-inflamed brain, typically represented by CD4^+^ T cells (CD45^+^CD4^+^), Ly6C^hi^ monocytes (CD45^hi^CD11b^+^Ly6C^hi^), and neutrophils (CD11b^+^Ly6G^hi^Ly6C^int^) (Fig. 2a and Supplementary Fig. S2a). A comparative analysis of the immune cell proportions revealed that, Rg1 treatment could selectively retard the brain accumulation of Ly6C^hi^ monocytes (Fig. 2b), while it exerted no significant effects on brain-infiltrated neutrophils (Fig. 2c) or CD4^+^ T cells (Fig. 2d). Ly6C^hi^ monocytes, also called proinflammatory monocytes, are myeloid cell subset that are reserved and primed in the periphery and traffick to the inflamed sites under chemotactic signals^24^. Of interest, flow cytometric analysis of Ly6C^hi^ monocytes in the periphery showed that Rg1 had no appreciable effects on the abundance of circulating Ly6C^hi^ monocyte pool (Fig. 2e), although it did effectively suppress their adaptive expansion in the spleen (Fig. 2f).

Since the retarded brain accumulation of Ly6C^hi^ monocytes was not associated with a direct inhibition on blood Ly6C^hi^ monocytes, we next sought to address whether Rg1 could exert a direct impact on the trafficking behavior of Ly6C^hi^ monocytes. To this end, we capitalized on an established FITC-labeling method to follow the track of blood-derived Ly6C^hi^ monocytes^12^. As shown in Supplementary Fig. S3a, FITC-microspheres injected into mice 20 h after the short-term depletion of peripheral monocytes by clodronate liposome were readily taken up by Ly6C^hi^ monocytes in the blood. No significant changes of the proportion of FITC-positive Ly6C^hi^ monocytes in the blood were observed among mice from different groups (Supplementary Fig. S3b). In the inflamed brain, as expected, a drastic infiltration of FITC-positive cells was observed, which were designated as Ly6C^hi^ monocytes from the periphery (Fig. 2g). Of note, the frequency of FITC^+^CD11b^+^Ly6C^hi^ monocytes in the brain was markedly decreased in Rg1-treated mice (Fig. 2h), which indicated that Rg1 could selectively inhibit the migration of peripheral Ly6C^hi^ monocytes to the inflamed brain.

### Rg1 represses the proinflammatory potential of Ly6C^hi^ monocytes

**Figure 3:**
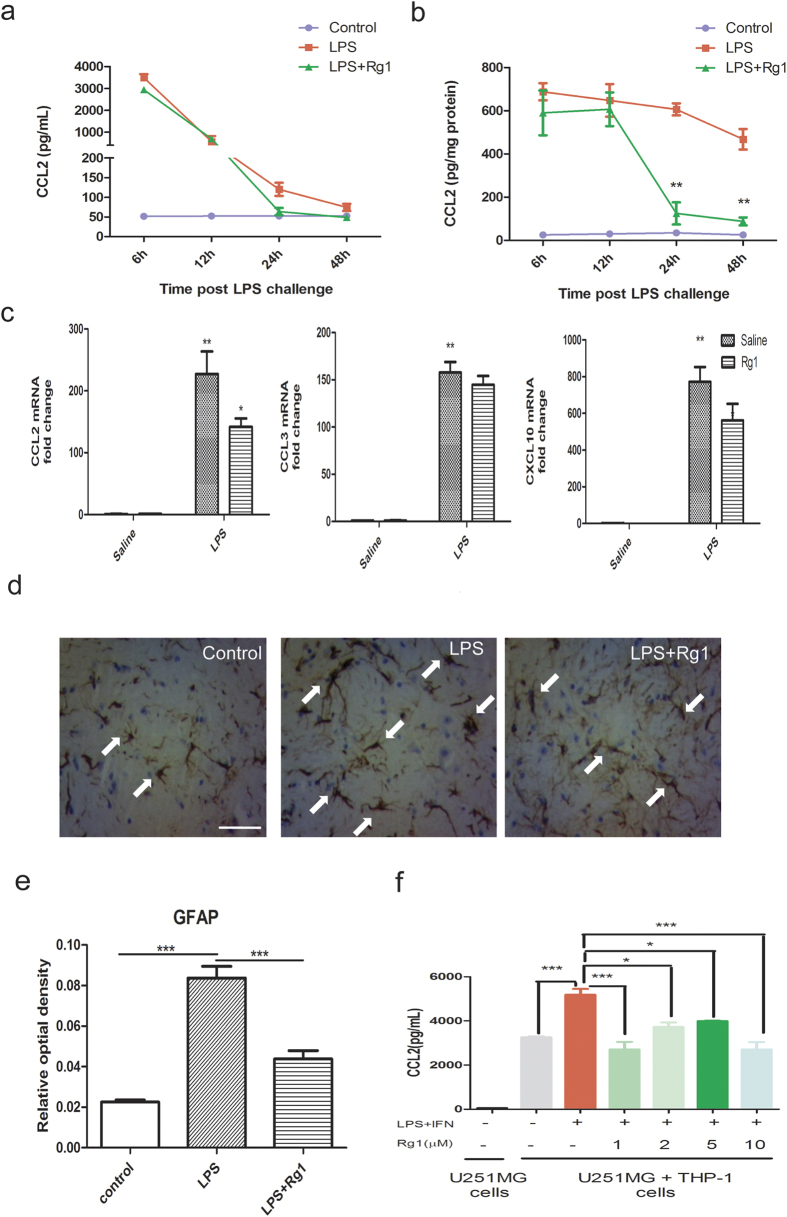
Inhibition of the proinflammatory potential of Ly6C^hi^ monocytes by Rg1. Mice received vehicle or Rg1 (20 mg/kg, i.p.) before central LPS challenge (3 μg). Mice were sacrificed at 6, 12, 24, 48 h after central LPS challenge, and the brain and blood were collected for analysis. (n = 4–5 mice per time point). (**a**) Time-dependent changes of CCL2 concentration in mice blood. (**b**) Time-dependent changes of CCL2 in mice brain. (**c**) Quantitative PCR analysis of the mRNA levels of CCL2, CCL3 and CXCL10 in the cerebral cortex of mice 24 h after i.c.v. LPS challenge. The ** symbols on the LPS-challenged control group indicates significant increase from saline control mice; while the * symbol on the Rg1-treated LPS mice indicates significant decrease from LPS-challenged control mice (n = 4–6 mice/group). (**d**) Representative immunohistochemical images of glial fibrillary acidic protein (GFAP) as marker of astrocytic activation. Brains from 3 mice in each group were processed for GFAP staining and 3 sections per mouse were included for quantification. Arrows indicate hyperactive astrocytes. Scale bars, 20 μm. (**e**) Statistical comparison of the GFAP expression using relative optical density. (**f**) The concentration of CCL2 in the supernatant of U251 MG (astrocyte cell line) - THP-1 (monocyte cell line) cell coculture system (2:1) at 24 h. THP-1 monocytes were pretreated with or without Rg1 (1, 2, 5, 10 μM), and then stimulated with LPS (1 μg/mL) and hIFNγ (1000 U/mL) for 48 h. Data represent mean ± s.e.m.^*^P < 0.05, ^**^P < 0.01,^***^P < 0.001.

CCL2 is the predominant chemoattractive ligand known to drive the activation and migration of Ly6C^hi^ monocytes^12^,^25^. The activation and recruitment of Ly6C^hi^ monocytes to the inflamed brain, in turn, could evoke CCL2 release to the circulation, thereby amplifying a self-perpetuating inflammatory circuit^25^,^26^. To further understand how Rg1 disrupts the brain accumulation of Ly6C^hi^ monocytes, we examined the effects of Rg1 on CCL2 dynamics in the brain and periphery. Rg1 had essentially no effects on the circulating CCL2 levels at several time points after central LPS challenge (Fig. 3a). Of particular interest, although no discernable changes on the early surge of cerebral CCL2 levels (6 h and 12 h post i.c.v. LPS) were observed, remarkable decreases in the prolonged upregulation of brain CCL2 could be found in Rg1-treated mice in the late phases (24 h and 48 h post i.c.v. LPS) (Fig. 3b). In consistence, quantitative PCR analysis also showed that Rg1 treatment could significantly blunt the transcriptional upregulation of CCL2 in the inflamed brain 24 h post LPS challenge, without notable effects on that of CCL3 or CXCL10 (Fig. 3c).

Astrocytes have been implicated as the major source of cerebral CCL2 after inflammatory activation^27^,^28^. Consistent with this fact, immunohistochemical (IHC) staining of astrocytic glial fibrillary acidic protein (GFAP), the marker of activated astrocytes, confirmed that astrocytic activation was effectively alleviated by Rg1 (Fig. 3d,e). In consideration of the fact that Rg1 could not directly act on astrocytes, we hypothesized that the proinflammatory capacity of Ly6C^hi^ monocytes may be targeted by Rg1 in the periphery, followed by a blunted astrocytic activation and disrupted brain-periphery signaling loop. To test this possibility, next we performed an *in vitro* co-culture assay using astrocytes (U251 MG) and monocyte (THP-1) cell lines. Compared with U251 MG cells cultured alone, a robust increase in CCL2 secretion was observed in the co-culture system, in line with the ability of monocytes to drive CCL2 release from astrocytes in the scenario *in vivo*. Of interest, this effect was even more dramatic when the THP-1 cells were primed by inflammatory activation prior to co-culture with U251 MG cells (Fig. 3f). Notably, Rg1-pretreated primed THP-1 cells failed to effectively evoke CCL2 secretion from co-cultured U251 MG cells, supporting the blunting effects of Rg1 on the proinflammatory potential of activated monocytes. Together, these *in vivo* and *in vitro* evidence strongly indicate that Rg1 is well-positioned in the periphery to specifically blunt the proinflammatory potential of Ly6C^hi^ monocytes.

### CCL2 signaling is involved in the anti-depressive effect of Rg1

**Figure 4:**
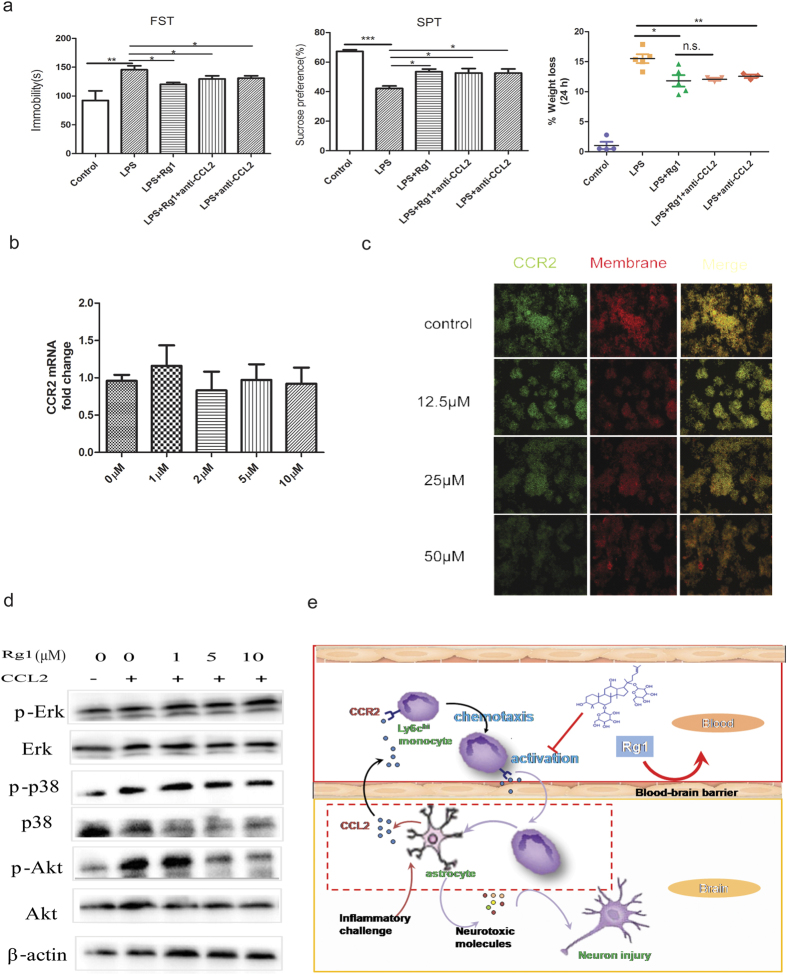
Antagonism of CCL2 signaling by Rg1. (**a**) Behavioral tests of mice in the forced swimming test (FST) and sucrose preference test (SPT) and body weight changes 24 h after central LPS challenge (3 μg). Mice received vehicle control, Rg1 (20 mg/kg, i.p.) or anti-CCL2 monoclonal antibody (100 μg/mice, i.p.) before central LPS challenge (3 μg).(**b**) Quantitative PCR analysis of CCR2 mRNA levels on cultured macrophages after the treatment with Rg1 at various concentrations (1, 2, 5, 10 μM). (**c**) Immunofluorescence analyses of the expression of CCR2 (green) in cultured macrophages. The cell membrane was visualized with Dil staining (red), and merged with CCR2 (yellow). No significant difference was observed on the expression of membrane CCR2. (**d**) Representative western blotting images for the MAPK (Erk/p-Erk, p38/p-p38) and PI3K-Akt (Akt/p-Akt) pathway in THP-1 cells. THP-1 cells were pretreated with culture medium with Rg1 (1, 5, 10 μM) or DMSO for 4 h and subjected to hCCL2 (10 nM) treatment for 4 min. (**e**) Schematic proposal of the mechanism by which Rg1 ameliorates neuroinflammation-associated depressive behavior from the periphery.

CCL2 signaling plays a key role in the activation and site-directed migration of monocytes^29^. To understand the potential role of CCL2 signaling in the anti-depressive effects of Rg1, we asked whether concomitant blockade of peripheral CCL2 signaling would influence the effect of Rg1 in LPS-challenged mice. We found that neutralization of peripheral CCL2 via a neutralizing monoclonal antibody (mAb) conferred a significant mitigation of the weight loss and depression-like behaviors induced by neuroinflammatory challenge (Fig. 4a), largely resembling those found in the Rg1-treatment group. In particular, co-treatment with CCL2 mAb and Rg1 produced no synergistic effects, which indicated an non-redundant role of CCL2 signaling in the anti-depressive mechanism of Rg1.

Based on the findings above, we next addressed how the CCL2 signaling was interrupted by Rg1. Results from the qPCR and immunofluorescence study indicated that Rg1 had no direct effect on the expression of CCR2, the membrane receptor of CCL2, in cultured monocytes (Fig. 4b,c). It is known that CCL2 binding to CCR2 on monocytes can elicit the activation of the mitogen-activated protein kinase (MAPK) and phosphatidylinositol 3-kinase (PI3K) pathways, which are critical determinants governing monocyte activation and migration^30^. We therefore asked whether Rg1 could modulate these kinase pathways downstream of CCL2-CCR2 interaction. Western blotting results showed that CCL2 triggered phosphorylation of p38 and Akt were markedly suppressed in the presence of Rg1 (1–10 μM), while the phosphorylation of Erk was only mildly inhibited by Rg1 (Fig. 4d). These results indicated that inhibition of CCL2-induced p38/MAPK and PI3K/Akt pathway activation could partially explain the inhibitory effects of Rg1 on proinflammatory monocytes. Taken the above evidence together, we propose that the blunting of the proinflammatory potential of Ly6C^hi^ monocytes in blood explain the paradox between the poor brain distribution and anti-depressive effects of Rg1 (Fig. 4e).

### Ly6C^hi^ monocytes contribute to behavioral deficits

**Figure 5:**
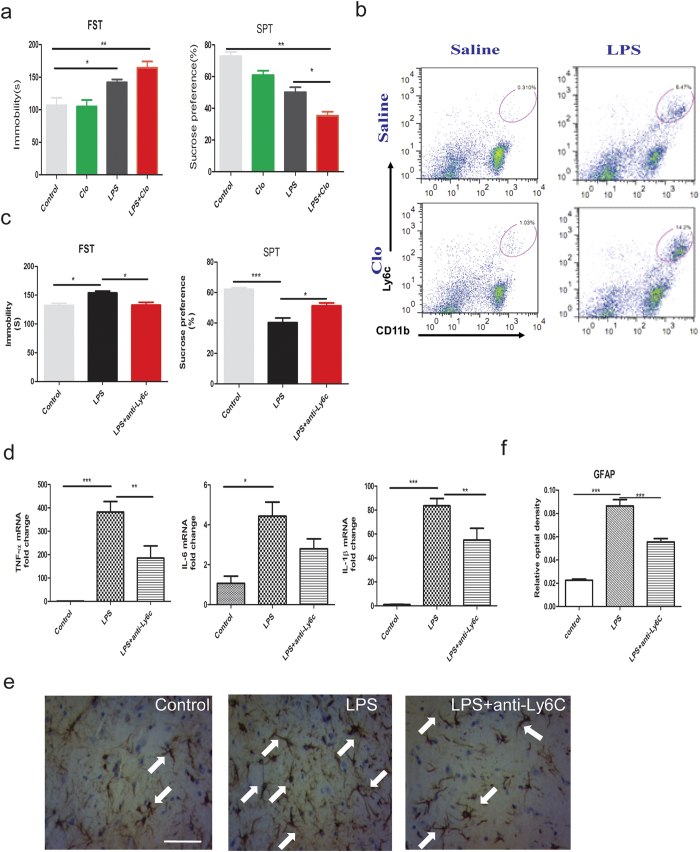
Ly6C^hi^ monocytes play a pathogenic role in neuroinflammation-associated behavioral deficits. (**a**) Assessment of the depression-like behaviors in forced swimming test and sucrose preference test in mice. Mice were injected with 200 μL of clodronate liposomes (Clo) via the tail vein, which could lead to a selective enrichment of circulating Ly6C^hi^ monocytes 72 h. Mice were then subjected to central LPS challenge (3 μg) and assessed for the behavioral changes 24 h later. (**b**) Flow cytometric analysis of brain infiltrated Ly6C^hi^ monocytes in LPS-challenged mice with or without prior enrichment. (**c**) Assessment of the depression-like behaviors of mice in the forced swimming test (FST) and sucrose preference test (SPT) 24 h after LPS challenge. Mice received anti-Ly6C monoclonal antibody (100 μg/mice, i.p.) to specifically block the activity of Ly6C^+^ monocytes, followed by central LPS challenge (3 μg) and behavioral assessment. (**d**) Quantitative PCR data for typical cytokines (TNF-α, IL-6, IL-1β) in the mice brain 24 h after central LPS challenge with or without Ly6C neutralization. The mRNA expression was normalized to β-actin. (n = 5–8 mice/group), n.s., not significant. (**e**) Representative immunohistochemical staining of GFAP as a reflection of the activation status of astrocytes. For each group, brains from 3 mice were processed for GFAP staining and 3 sections per mouse were included for quantification. Arrows indicate hyperactive astrocytes. Scale bars, 20 μm. (**f**) Statistical comparison of the GFAP expression using relative optical density. Data represent mean ± s.e.m. ^*^P < 0.05, ^**^P < 0.01, ^***^P < 0.001.

The dampening of Ly6C^hi^ monocytes in the periphery by Rg1 underlying its anti-depressant benefits hinted to a potential pathogenic role of Ly6C^hi^ monocytes in neuroinflammation-associated behavioral deficits. To provide direct evidence, peripheral Ly6C^hi^ monocytes were specifically enriched or neutralized and the behavioral outcomes were evaluated respectively. Firstly, we capitalized on a previous report that peripheral blood monocytes, transiently depleted with clodronate liposomes, were replaced within 18 to 24 h by bone marrow mononuclear cells that were heavily enriched by the CD11b^+^Ly6C^hi^ subset (Supplementary Fig. S4a). Using this method, we found that enrichment of blood Ly6C^hi^ monocytes clearly exacerbated the severity of depression-like behavior of mice (Fig. 5a), with a heightened central accumulation of Ly6C^hi^ monocytes (Fig. 5b and Supplementary Fig. S4b). Next, we specifically depleted Ly6C^hi^ monocytes with an anti-Ly6C mAb, which was previously validated to blunt the proinflammatory potential of peripheral Ly6C^hi^ monocytes^31^. Behavioral tests revealed that treatment with anti-Ly6C antibody lead to an improvement in the depressive symptoms (Fig. 5c). Quantitative PCR results disclosed that the transcriptional upregulation of typical proinflammatory cytokines was also effectively suppressed after antagonism of Ly6C^hi^ monocytes in blood (Fig. 5d). Moreover, GFAP staining showed that dampening Ly6C^hi^ monocytes with anti-Ly6C antibody could significantly attenuate astrocytic activation (Fig. 5e,f). Collectively, these results suggest that peripheral Ly6C^hi^ monocytes could be specifically manipulated to remotely regulate neuroinflammation and associated behavioral changes.

### Dampening Ly6C^hi^ monocytes attenuates stress-induced depression-like behavior

**Figure 6:**
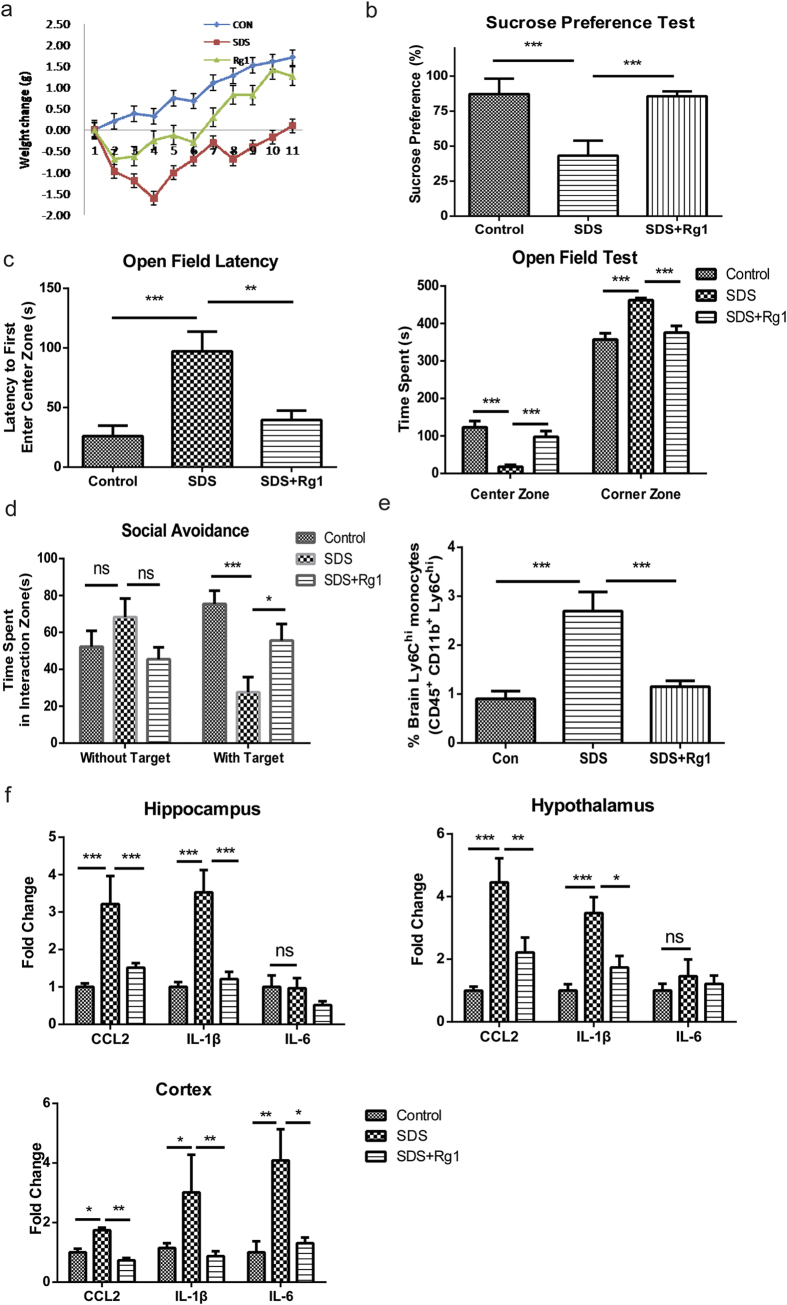
Dampening Ly6C^hi^ monocytes alleviates social defeat stress-induced behavioral symptoms and neuroinflammation in mice. In chronic stress-induced depression paradigm, mice were subjected to a 10-day social defeat stress (SDS) to induce depression-like behaviors. Rg1 (20 mg/kg, i.p., once daily) was administered for 28 days before and throughout the 10-day SDS paradigm. At the end of behavioral tests, mice were sacrificed and tissue samples were collected for analysis. (**a**) Daily body weight changes of mice compared to the baseline level during the whole course of chronic stress. (**b**) Sucrose preference test to assess the depression-like behaviors of mice. (**c**) Open-field test to assess the anxiety-like behaviors of mice. (**d**) Social interaction test to assess the social avoidance behavior of mice. (n = 10–13 mice/group). (**e**) Quantitative comparison of the brain infiltrating Ly6C^hi^ monocytes by flow cytometric analysis. (**f**) Quantitative PCR analysis of typical inflammatory mediators (CCL2, IL-6, IL-1β) in the hippocampus, hypothalamus and cortex (n = 6–8 mice/group). ns, no significant difference; SDS, social defeat stress. Data represent mean ± s.e.m. ^*^P < 0.05, ^**^P < 0.01, ^***^P < 0.001.

Bidirectional communication between the brain and immune system has been increasingly implicated in the development of behavioral symptoms in relation to stressful events in the clinical scenario^32^,^33^,^34^. The pathological role of Ly6C^hi^ monocytes in driving neuroinflammatory disturbance and associated depression-like behavior therefore prompted us to investigate whether dampening them in blood could protect against psychosocial stress-induced behavioral symptoms. Importantly, the availability of Rg1 as a small-molecule inhibitor of Ly6C^hi^ monocytes provided us with a well-suited tool to address this question. A chronic social defeat stress (SDS)-induced mice depression model that recapitulates typical symptoms of depressive disorders in the clinic was used. Rg1-treated SDS mice exhibited an earlier and more pronounced body weight recovery following sharp weight losses during the initial social attacks (Fig. 6a). Notably, Rg1 treatment (20 mg/kg, i.p., once daily), to a large extent, successfully relieved the anhedonia and anxiety-like behaviors in socially-defeated mice, as reflected by sucrose consumption preference and prolonged durations in the center zone (Fig. 6b,c and Supplementary Fig. S5a). Moreover, in the social interaction test, Rg1-treated mice exhibited much better post-attack social interaction performances than saline-treated counterparts when faced with a caged aggressor (Fig. 6d and Supplementary Fig. S5b). Flow cytometric analysis of the cell infiltrates in the mice brain further indicated that chronic SDS induced a significant increase in infiltrating Ly6C^hi^ monocytes to the brain. In contrast, the percentage of brain infiltrated Ly6C^hi^ monocytes was significantly lower in Rg1-treated mice (Fig. 6e and Supplementary Fig. S5c), in line with the dampening of this cell subpopulation. These results convincingly demonstrated that inhibiting peripheral Ly6C^hi^ monocytes in the periphery conferred anxiolytic and anti-depressive effects in the context of chronic psychosocial stresses. Consistent with the pro-inflammatory effects of Ly6C^hi^ monocytes, we observed that the behavioral benefits of chemical dampening Ly6C^hi^ monocytes were accompanied by significant amelioration of the inflammatory disturbance in several brain regions including the hippocampus, hypothalamus and cortex (Fig. 6f). In addition, a significant decrease of spleen swelling and blood CCL2were found in Rg1-treated mice (Supplementary Fig. S5d,e), which indicated attenuated peripheral immune disturbances in the context of psychosocial challenges.

## Discussion

It is increasingly appreciated that the immune system plays an important role in shaping the psychiatric state, but it remains largely unclear how this link could be therapeutically harnessed to treat mental and behavioral disturbances. Targeted modulation of neuroimmune mediators in psychiatric disorders will inform the pathological mechanisms and development of treatment or prevention strategies. In this work, we identify that Ly6C^hi^ monocytes in blood can serve as a viable target to counteract the mental disturbances induced by neuroinflammation or psychosocial stress. This is attributed to the discovery of Rg1 as, what is to our knowledge, the first chemical inhibitor of Ly6C^hi^ monocytes. In line with the increasing understanding of depression as a systems disorder, our findings suggest that Ly6C^hi^ monocytes in blood can be targeted for remote intervention of neuroinflammation and stress-associated behavioral deficits, without the need to cross the blood-brain barrier.

Our previous efforts to elucidate the working mechanism for ginsenosides suggest a potential periphery-to-brain action mode^18^,^19^. Although current studies largely seek to find a neuro-centric explanation for the CNS benefits of Rg1 and other ginsenoside compounds^22^,^23^,^35^, here we provide convincing evidence that specific blunting of the proinflammatory properties of Ly6C^hi^ monocytes in the periphery is the novel model of action of Rg1. The pharmacokinetic and tissue distribution results of Rg1 in the neuroinflammatory mice model dissociate its anti-depressive effects from a direct brain action. Support for this notion further comes from the finding that the feedback release of CCL2 from the brain, rather than the early surge after central LPS challenge, was abrogated by Rg1. This is further evidenced by a significant inhibition of astrocytic activation by Rg1, which contributes to the disruption of a self-perpetuating loop of neuroinflammatory disturbance. Of interest, Rg1 had no direct effect on the percentage of circulating Ly6C^hi^ monocytes, but effectively decreased their abundance in the spleen reservoir. This discrepancy underscores the need to dissect the exact site of action for Rg1, especially given the pleiotropic activities of this natural compound. In recent years, several lines of evidence uncover that the spleen is a critical reserving site for primed monocytes^5^,^9^, which are shown to have increased potential for inflammatory signaling. Therefore, it is interesting to consider that Rg1 may act on the spleen to suppress the priming of monocytes in response to neuroinflammatory or psychosocial stressors. As for the molecular mechanisms, we find that the MAPK and PI3K/Akt signaling pathway downstream of CCL2-CCR2 interaction may explain the dampening effects of Rg1 on monocyte activation. Future in-depth proteomic profiling of the binding protein of Rg1 is expected to decipher the molecular basis of this effect.

The availability of Rg1 as a peripheral-restricted compound provides an essential probe to identify the Ly6C^hi^ monocytes as potential anti-depressive target. Ly6C^hi^ monocytes are versatile immune cells implicated in a variety of inflammatory disorders such as multiple sclerosis^36^, spinal cord injury^15^, and heart ischemia^37^; however, their exact role in depression pathogenesis remains elusive. In our study, we observed a selective inhibitory effect of Rg1 on the recruitment of Ly6C^hi^ monocytes to the inflamed brain. Specific augmentation and antagonism studies collectively confirmed the pathogenic role of Ly6C^hi^ monocytes in aggravating neuroinflammatory disturbance. Also, blockade of Ly6C^hi^ monocytes conferred marked resilience against psychosocial stress-induced behavioral deficits. These lines of evidence collectively point to a pathogenic role of Ly6C^hi^ monocytes in neuroinflammation and stress-associated depression. This finding, together with a recent report^9^, strengthens the theory that the peripheral immune system could be manipulated for neuropsychiatric regulation in the CNS. In particular, the anti-depressive effect of Ly6C^hi^ monocytes dampening was accompanied by a successful inhibition of astrocytic activation, which suggests that the reinforcing process of neuroimmune disturbance in the brain can be disrupted by agents in the periphery. In future studies, the glial phenotype and neurogenesis parameters in key brain regions underlying emotional control such as the prefrontal cortex and amygdala should be profiled to better understand the neuroimmune correlates of peripheral Ly6C^hi^ monocytes.

In the clinical scenario, chronic psychosocial stress has been established as an important trigger of a plethora of mental diseases. Chronic stress is recently found to promote the proliferation of hematopoietic stem cells, leading to higher levels of circulating inflammatory leukocytes in humans^5^. Herein, we provide the novel experimental evidence that peripherally dampening of Ly6C^hi^ monocytes could become a promising strategy to protect against chronic stress-associated behavioral deficits as well as the central inflammatory disturbance. From the point of clinical translation, the results of our study indicate that combating neuroimmune disturbance at the systems level can serve as a novel strategy to attenuate the deleterious effects of chronic stress. Recently, of interest, it was reported that blocking the brain recruitment of Ly6C^hi^ monocytes prevented repeated social defeat-induced anxiety in mice^9^,^38^. Also, adoptive transfer of the lymphocytes from mice undergoing chronic social defeat stress is shown to protect against the deleterious effects of stress on the naïve mice and reduce stress-induced pro-inflammatory cytokine levels in the blood^39^. Taken together, these emerging findings highlight the promise of manipulating the peripheral immune system to protect against the deleterious effects of psychosocial stresses, the clinical prospect of which warrants further investigations.

The identification of Rg1 as a peripherally-restricted inhibitor of Ly6C^hi^ monocytes may provide a new framework for understanding the central benefits of ginseng, an herbal medicine that has been used for thousands of years. For the perspective of reverse pharmacology, dissecting the mechanisms underlying the well-validated clinical efficacy is an approach for discovering novel therapeutics. Indeed, our work has implications for innovating treatment strategies of depression with periphery-targeting drugs. Although long persistent efforts in the discovery of druggable targets for CNS diseases are largely brain-biased, our findings underscore the necessity to look beyond the brain to combat the mental disturbance associated with inflammatory disturbance and stressful events. Targeting immune disturbance in the brain has long been pursued for CNS drug discovery. However, the dilemma between insufficient brain penetration^40^ and undesirable CNS side effects^41^,^42^ are typical challenges that impede the conventional CNS-biased drug discovery paradigm. Given this caveat, the novel acting mode of Rg1 encourages the exploration of compounds that work peripherally to control central disturbances. Future studies are therefore warranted to identify and validate the direct molecular target of Rg1 in dampening the proinflammatory activities of peripheral inflammatory monocytes. Meanwhile, Rg1 can be used as a lead to inspire the design and development of chemical drugs targeting proinflammatory monocytes. As Ly6C^hi^ monocytes also play key pathogenic roles in several inflammatory disturbances both in and outside the brain, one may explore more therapeutic values of specific chemical inhibitors of Ly6C^hi^ monocytes.

In summary, we describe several lines of evidence illustrating the pathogenic role of Ly6C^hi^ monocytes in depression, which is amenable to targeted blockade at the periphery. Importantly, the characterization of Rg1, a natural compound that is not brain penetrant, as a selective chemical inhibitor of Ly6C^hi^ monocytes provides a novel tool to address the pathophysiological role of this cell population. From a therapeutic standpoint, these findings substantiate the need to take a holistic approach to depression, and suggest that targeting the peripheral immune cells like Ly6C^hi^ monocytes may open a new avenue to achieve remote control of depression.

## Materials and Methods

### Reagents

Ginsenoside Rg1 (purity > 98%) was purchased from Jilin University (Changchun, China) and dissolved in sterile saline solution for administration. Purified CCL2 and IFN-γ were purchased from Peprotech (Rocky Hill, NJ, USA). Antibodies for the flow cytometric analysis and the targeted blockade of mouse CCL2 and Ly6C were all purchased from Biolegend (San Diego, CA, USA). Antibodies for the western blotting were purchased from Cell Signaling Technology (Danvers, MA, USA). Percoll and lympholyte-Mammal separation medium were purchased from GE Healthcare (Sweden) and Cedarlane Laboratories (Hunby, Ontario, Canada), respectively. Agents for the qRT-PCR analysis were purchased from Takara (Takara, Kyoto, Japan).

### Animals and treatment

Male C57BL/6 mice, used at 6–8 week of age, were obtained from the China Academy of Military Medical Sciences (Beijing, China). Male CD-1 retired breed mice (8–9 months) were obtained from the Vital River Laboratories (Beijing, China). The animals were housed under standard laboratory conditions, with constant temperature (22 °C), 12 h day/night cycle (lights from 8 am to 8 pm) and food/water *ad libitum*. All animal procedures were performed in strict accordance to the Institutional Animal Research Committee guidelines and approved by the Animal Ethics Committee of China Pharmaceutical University. All efforts were made to minimize animal suffering and reduce the number of animals used.

In neuroinflammation-induced depressive mice model, Rg1 was administered intraperitoneally (i.p.) once daily to mice at a dosage of 20 mg/kg for 4 days before and right after the neuroinflammatory challenge with LPS. In the social defeat stress paradigm, Rg1 (20 mg/kg, i.p.) administration started 4 days before the initiation of daily social stress and continued throughout to the end. (See also Supplementary Fig. S6).

### Induction of depression-like behavior

To induce neuroinflammation, mice were anesthetized with sodium pentobarbital (50 mg/kg) and mounted onto the stereostatic apparatus. LPS (*Escherichia coli* serotype 055:B5, 3 μg/3 μL; Sigma-Aldrich) or vehicle (saline) was microinjected into the lateral ventricle at a rate of 0.5 μL/min using a microinjection pump (Harvard Apparatus, MA, USA), which elicited very mild damage to the brain architecture^43^,^44^. The coordinates for the lateral ventricle are 1.0 mm lateral, 0.5 mm posterior and 2.5 mm ventral from the bregma. Control animals received an iso-volumetric injection of sterile saline.

Chronic social defeat stress was conducted according to the method previously reported^45^. Briefly, before the experiment, retired CD-1 breeder mice were screened for 3 days to select qualified aggressive mice which were then housed singly in one side of cage equipped with perforated divider to habituate to their colony. During daily social confrontation (between 16:00 pm to 18:00 pm), a C57BL/6 mouse was exposed to an aggressive CD-1 mouse for 5–10 min to receive sustained defeat. Submissive/subordinate behavior included upright posture, fleeing, and crouching of the resident mice and the total aggressive interactions were 2–5 min in each paradigm. After that, the “intruder” was transferred to the other side of the divider and housed overnight. The C57BL/6 mice were exposed to novel CD-1 aggressors for 10 consecutive days. Care was taken to maximally avoid any physical lesions on the “defeated” mice. Control mice were left in their home cages undisturbed.

### Behavioral tests

Prior to each behavioral test, mice were acclimatized to the experimental room for at least 1 hour. (See also Supplementary Fig. S6).

The forced swimming test was initiated by placing mice in an inescapable cylinder (diameter 24 cm, height 30 cm) containing 12 cm of water maintained at 25 °C. Forced swimming was video-recorded for 6 min. Immobility was defined as motionless floating in the water with minimum movements. The duration of swimming, climbing, and immobility was determined by a trained observer who was blind to the experimental treatments.

For the tail-suspension test, mice were suspended by their tails with adhesive tape and video-recorded for 6 min. Immobility was defined as motionless state without the intension to escape. For the sucrose preference test, mice were acclimated to two bottles (one filled with drinking water and the other with 1.5% sucrose water) for one day. On the day of experiment, mice were given a free choice between two bottles, one with 1.5% sucrose solution and another with tap water for 24 h. The position of the bottles was switched every 6 h to avoid possible effects of side preference in drinking behavior. The consumption of water and sucrose solution was estimated by weighing the bottles before and after the test.

Anxiety-like behaviors was tested in an open field test. Generally, mice were put into the arena of a square plastic box (42 × 42 cm) to record their movements. Adopting an EthoVision automatic tracking system (Noldus, Netherland), the track of the last 8 min was plotted and analyzed to measure the time spent in the “center zone” (30 × 30 cm) and “corner zones”.

For social interaction test, the reaction of defeated mice towards the aggressor were recorded and measured to determine the degree of their social avoidance behaviors. During the first stage, a C57BL/6 mouse was introduced to an arena with a small empty cage (12 × 8 cm) and its activity was recorded for 150 s. Next, the mouse was removed and a novel aggressor was put into the cage as a “target”. During the second stage, the same mouse was again introduced and its reaction towards the new “target” was recorded for another 150 s. Through the comparison of mice duration in “interaction zone” (an arena 8 cm wide surrounding the cage) between the two stages, the social avoidance degree of the mouse was measured. The mice were subjected to behavioral tests 24 h after the central LPS challenge or at the end of the social defeat stress paradigm.

### Immunohistochemistry

Detailed procedures for the immunohistochemical staining and analysis of GFAP are provided in the Supplementary information.

### Isolation of leukocytes from mouse brain, blood and spleen

Mice designated for flow cytometry analysis were anesthetized and then perfused transcardially with ice-cold PBS. The brain, blood and spleen were carefully collected and processed for the isolation of target cells as specified in the Supplementary information.

### Flow cytometry

Staining of cell surface antigens was performed according to the manufactures and the procedures are specified in the Supplementary information.

### Labeling, enrichment and functional blockade of Ly6C^hi^ monocytes

Mice received 200 μL clodronate liposomes intravenously and 20 h later, 0.25 mL of 0.5 μm Fluoresbrite fluorescein isothiocyanate (FITC)-dyed (YG) plain microspheres (Polysciences, USA) were injected intravenously to selectively label activated monocytes. To specifically enrich Ly6C^hi^ cells, mice were injected intravenously with 0.25 mL clodronate liposomes (Formumax, USA) or PBS liposomes, and the peripheral monocyte pool was replenished within 24 h. For the functional blockade of Ly6C^hi^ monocytes, mice were intraperitoneally administered with anti-mouse Ly6C antibody (Clone HK 1.4, 100 μg) or isotyoe controls (Rat IgG2c, κ isotype control, 100 μg).

### CCL2 neutralization

For CCL2 neutralization, animals received either i.p. injections of anti-mouse CCL2 antibody (Clone 2H5, 100 μg) or isotype controls (Armenian Hamster IgG isotype control, 100 μg) at 2 h after i.c.v. injection of LPS.

### Quantitative real-time PCR

Total RNA isolation, reverse transcription and real-time qPCR reactions were carried out with standard protocols as specified by the manufacturer. The primer sequences are listed in the Supplementary information.

### *In vitro* co-culture of U251 MG and THP-1 cells

The human leukemia cell line THP-1 cells were cultured in RPMI-1640 supplemented with 10% fetal bovine serum. The human astrocytoma cell line U251 MG was cultured in DMEM with 10% fetal bovine serum, 1% L-glutamine and 100 U/mL of penicillin/streptomycin. All the cell lines were maintained under 5% CO_2_ in 37 °C incubator. Before setting up the co-culture process, THP-1 monocytes were stimulated with 1 μg/mL LPS and 1000 U/mL hIFN-γ for 48 h. After a through wash with blank RPMI-1640 for three times, the THP-1 cell suspensions were added to the U251 MG cells and co-cultured for another 24 h. The supernatant was collected and the CCL2 release was determined by ELISA (Biolegend, USA).

### Western blotting

THP-1 cells were cultured to reach 80% confluence before experiment. Cells were pretreated with Rg1 for 4 h, and then stimulated with hCCL2 (10 nM, Peprotech, USA) for 3 min. The cells were quickly centrifuged, thoroughly washed with cold PBS, and 100 μL of ice-cold lysis buffer containing 1 mM PMSF and phosphatase inhibitors were added. The separation and detection of targeted protein bands were carried out following standard protocols specified in the Supplementary information.

### Quantitative determination of Rg1 in mice plasma and tissues

The concentrations of Rg1 in mice brain, plasma, liver and spleen were determined by a validated liquid chromatography-mass spectrometry (LC-MS) method as previously reported by our group^19^.

### LC-MS/MS determination of neurotransmitter and their metabolites

The concentrations of dopamine, serotonin, 5-hydroxyindoleacetic acid, kynurenic acid and 3-hydroxykynurenine in mice brain were determined by a validated LC-MS/MS method as specified in the Supplementary information.

### Statistical analysis

Statistical difference between two groups was analyzed using Student’s t-test. Multiple comparisons were statistically analyzed with one-way analysis of variance (ANOVA) followed by Tukey multiple comparison *post hoc* analysis. *P* < 0.05 was considered statistically significant.

## Additional Information

**How to cite this article**: Zheng, X. *et al.* Chemical dampening of Ly6C^hi^ monocytes in the periphery produces anti-depressant effects in mice. *Sci. Rep.* **6**, 19406; doi: 10.1038/srep19406 (2016).

## Supplementary Material

Supplementary Information
