# Supplementary material for: Chemical dampening of Ly6Chi monocytes in the periphery produces anti-depressant effects in mice
Source: Sci Rep. 2016 Jan 19;6:19406. doi: 10.1038/srep19406 (PMC4725984; doi:10.1038/srep19406)
Supplement: Supplementary Information [file srep19406-s1.pdf]

Supplementary Information

Chemical dampening of Ly6C^{hi} monocytes in the periphery produces anti-depressant effects in mice

Xiao Zheng[1,2,5], Sijing Ma[1,3,5], An Kang[4], Mengqiu Wu[1], Lin Wang[1], Qiong Wang[2], Guangji Wang[1,*] & Haiping Hao[1,*]

[1]State Key Laboratory of Natural Medicines, China Pharmaceutical University, Nanjing 210009, Jiangsu, China

[2]Department of Pharmacy, Nanjing University of Chinese Medicine Affiliated Hospital, Nanjing 210029, Jiangsu, China

[3] Department of Pharmacy, Affiliated Hospital of Hunan Academy of Chinese Medical Sciences, Changsha 410000, Hunan, China

[4] School of Pharmacy, Nanjing University of Chinese Medicine, Nanjing 210023, Jiangsu China

[5]Co-first author

*Correspondence: hhp_770505@hotmail.com (H.H.), or guangjiwang@hotmail.com (G.W.)

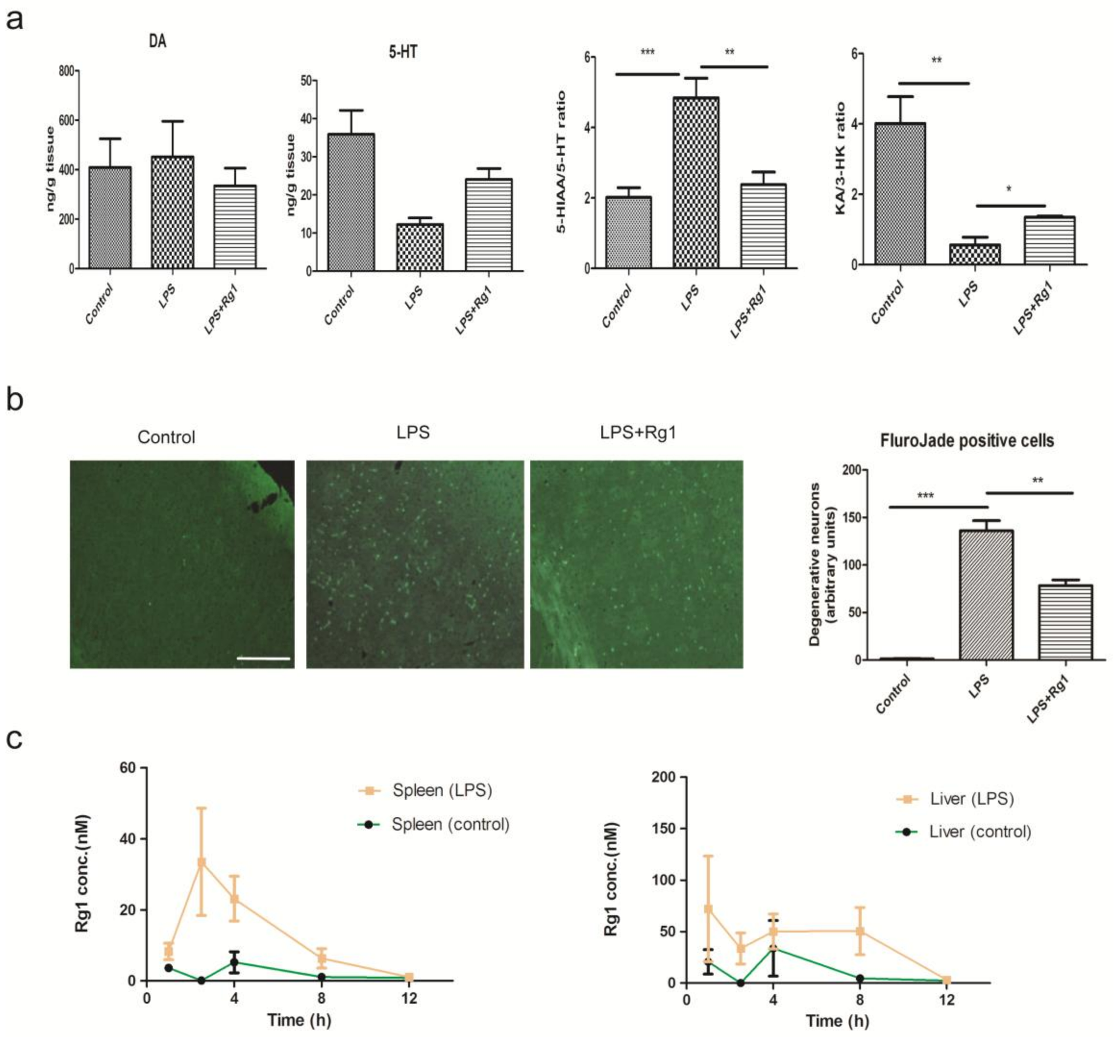

Supplementary Figure S1. **Rg1 ameliorates neuronal injury without entering the brain.** (**a**) Determination of typical neurotransmitters and neuroactive metabolites in mouse brain by LC-MS/MS. DA, dopamine; 5-HT, serotonin; 5-HIAA, 5-hydroxyindoleacetic acid; KA, kynurenic acid; 3-HK, 3-hydroxykynurenine. (**b**) Representative Fluoro-Jade B staining of neuronal death in mice brain. Light green cells indicate degenerative or dead neurons. Scale bar, 50 μm. (**c**) The concentration of Rg1 in mice spleen and liver 1, 2.5, 4, 8, 12 h after intraperitoneal administration. Mice received a central challenge of LPS (3 μg, 3 μL) or equal volume of saline. Data are expressed as mean ± s.e.m. n = 4-6 mice each treatment group. $P < 0.05$, ** $P < 0.01$, *** $P < 0.001$.

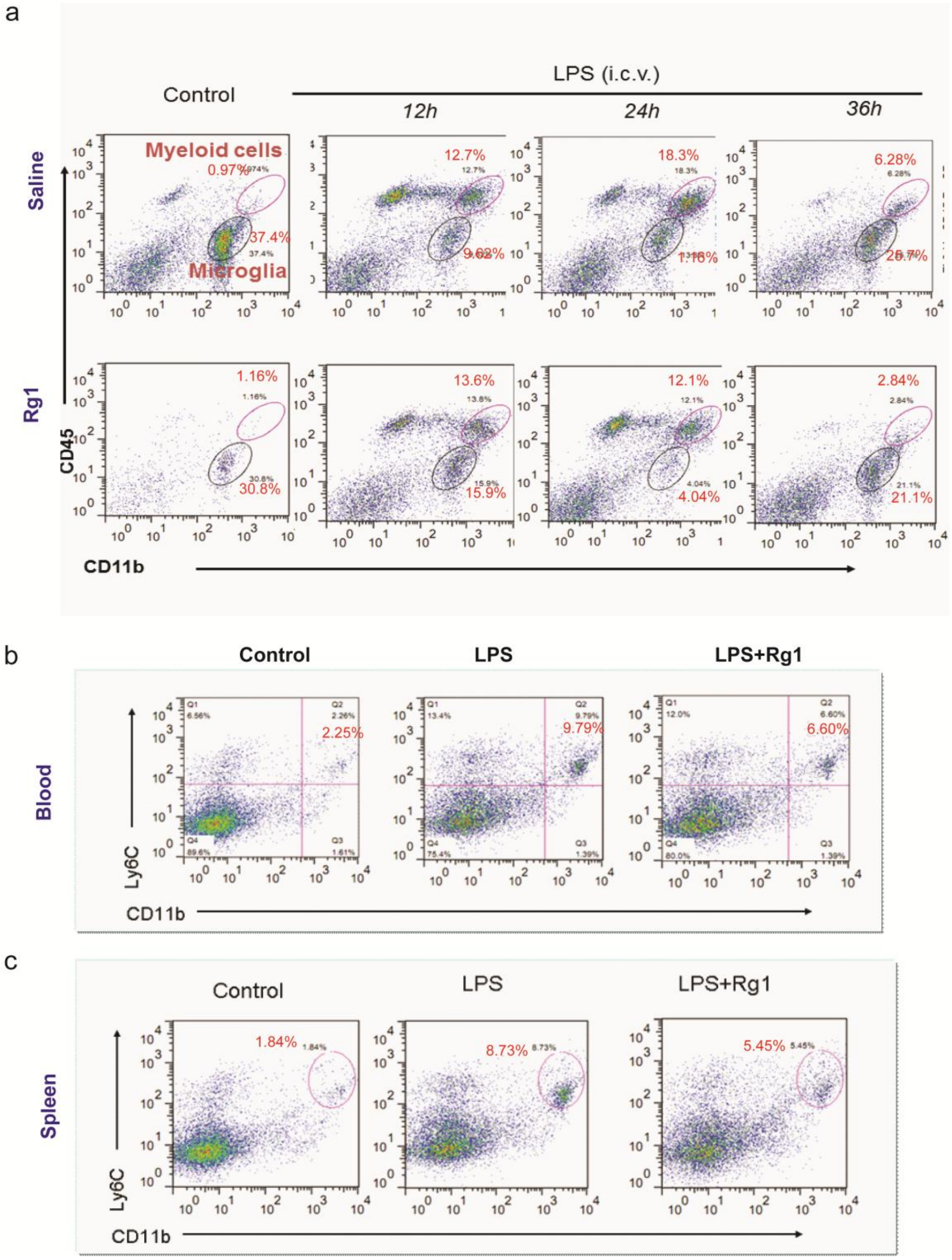

Supplementary Figure S2. **Effect of Rg1 on the brain infiltration of peripheral immune cells.** Mice received vehicle or Rg1 (20 mg/kg, i.p., once daily for 4 consecutive days) before an intracerebroventricle LPS challenge. (**a**) Kinetic study of the brain infiltrated myeloid cells 24 h after LPS challenge in mice treated with (lower panel) or without (upper panel) Rg1. The gates indicate myeloid cells ($CD11b^{+}CD45^{hi}$) and microglia ($CD11b^{+}CD45^{int}$) populations, respectively. (**b**) Representative flow cytometric plots of $Ly6C^{hi}$ monocytes ($CD11b^{+}Ly6C^{hi}$) in blood after the induction of neuroinflammation by LPS. (**c**) Representative flow cytometric plots of $Ly6C^{hi}$ monocytes ($CD11b^{+}Ly6C^{hi}$) in spleen after the induction of neuroinflammation by LPS.

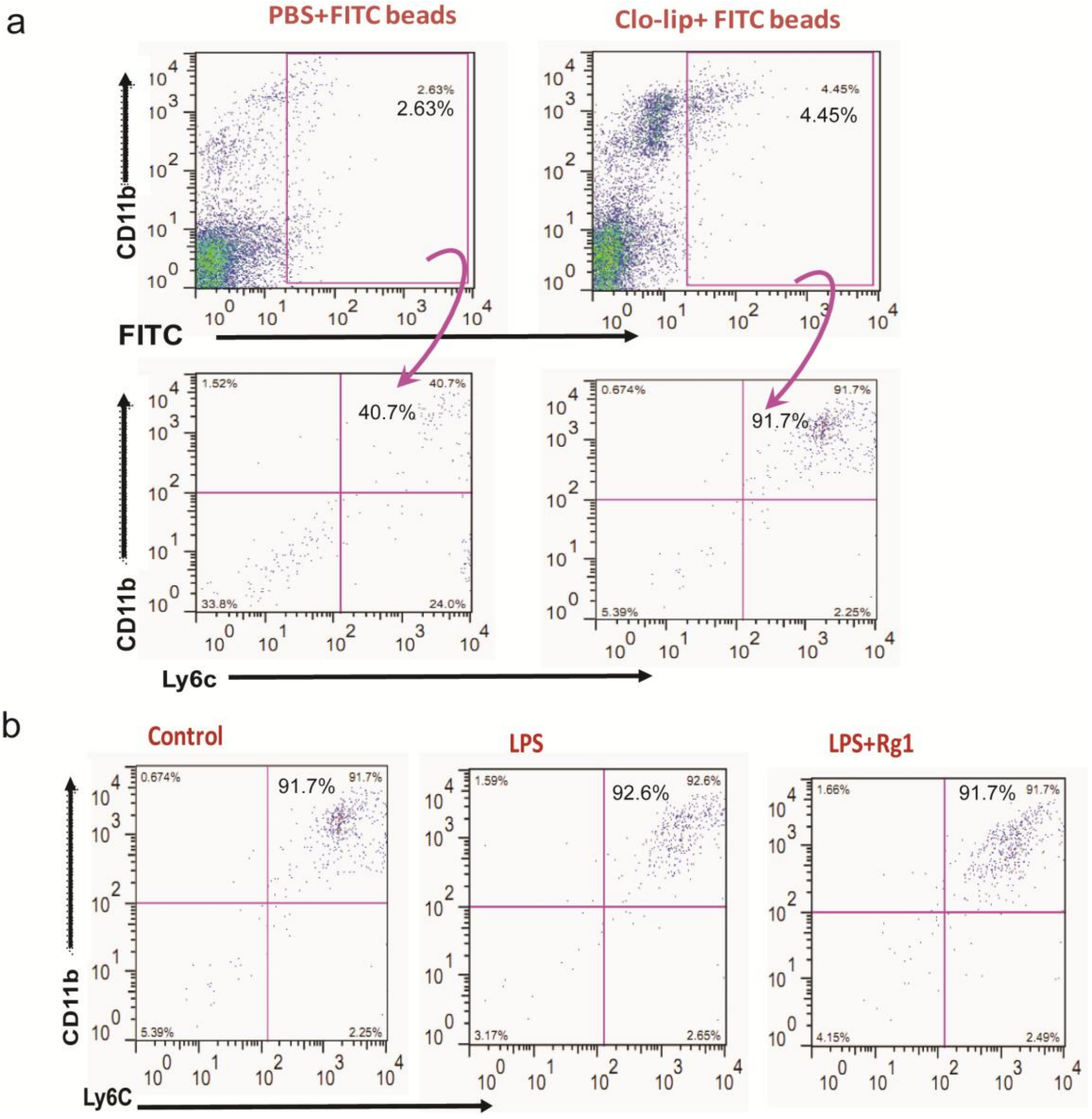

Supplementary Figure S3. **Effect of Rg1 on the brain recruitment of FITC-labled Ly6C^{hi} monocytes in mice.** The Ly6C^{hi} monocytes from the periphery were selectively labeled with FITC microspheres, and were thereafter gated by FITC^{+}CD11b^{+}Ly6C^{hi}. (**a**) Labeling of circulating Ly6C^{hi} monocytes with FITC with a ratio up to 91.7% . (**b**) Flow cytometric analysis of blood Ly6C^{hi} monocytes after FITC labeling.

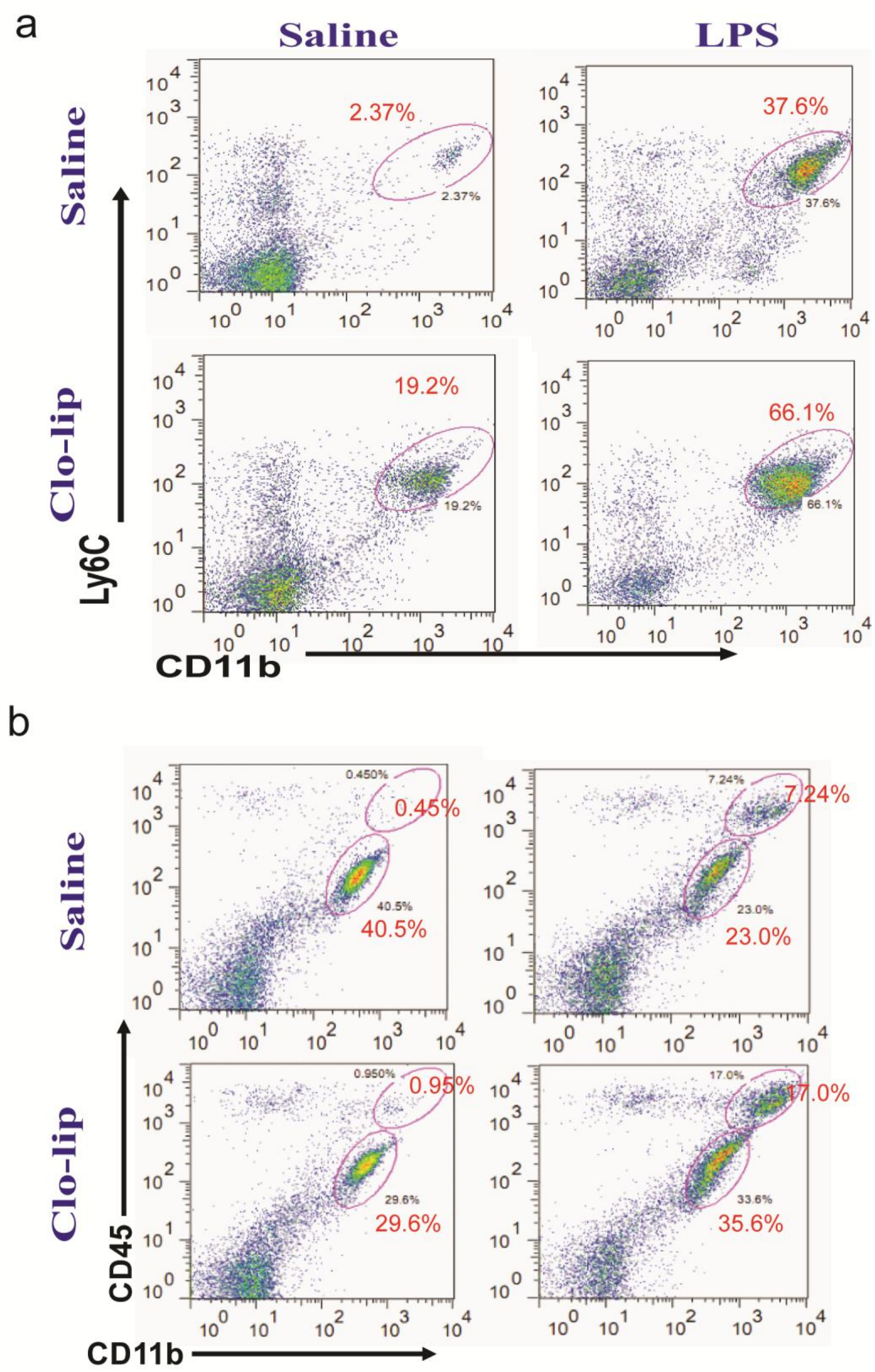

Supplementary Figure S4. **Brain infiltration of Ly6C^{hi} monocytes after specific enrichment of this cell population in blood.** (**a**) Flow cytometric analysis of Ly6C^{hi} monocytes (CD11b^{+}Ly6C^{hi}) in the blood after the challenge of central LPS. (**b**) Flow cytometric analysis of infiltrated myeloid cells (CD11b^{+}CD45^{hi}) and microglia cells (CD11b^{+}CD45^{int}) in the inflamed brain after Ly6C^{hi} monocytes enrichment. Clo-lip, clodronate liposomes.

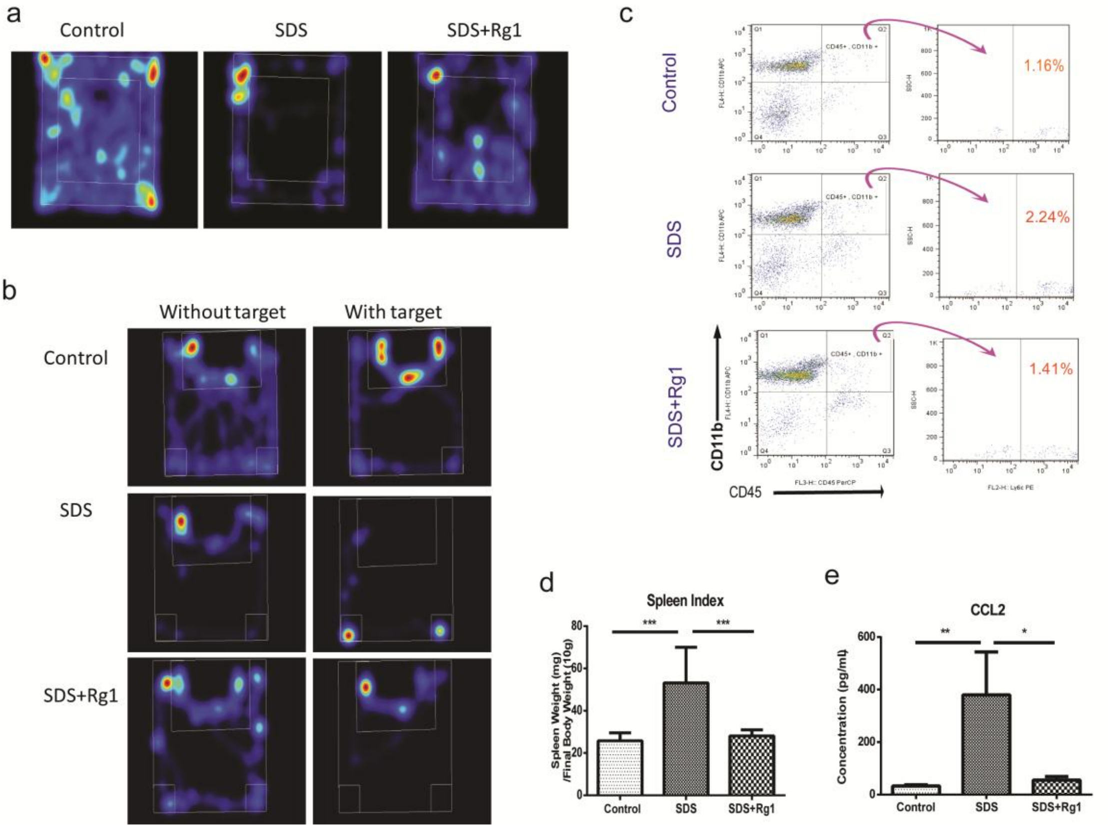

Supplementary Figure S5. **Effect of Ly6C^{hi} monocyte dampening on the behavior and peripheral disturbances in mice subjected to chronic social-defeat stress.** (**a**) Representative heat map images of mice movement tracks in the open field test. The large and small box indicate the area of the “open field” and “center zone”, respectively. (**b**) Representative heat map images of the mice in the social avoidance test without or with a “target”. The large box indicate the area of the test field, in which the small upper and lower boxes indicate the “center zone” and “corner zones”, respectively. (**c**) Flow cytometric plots of brain infiltrated Ly6C^{hi} monocytes. Numbers indicate the percent of Ly6C^{hi} monocytes. (**d**) The ratio of mice spleen weight to the whole body weight at the end of behavioral study. (**e**) Serum CCL2 level in mice determined by ELISA. SDS, social defeat stress.* $P < 0.05$, ** $P < 0.01$, *** $P < 0.001$.

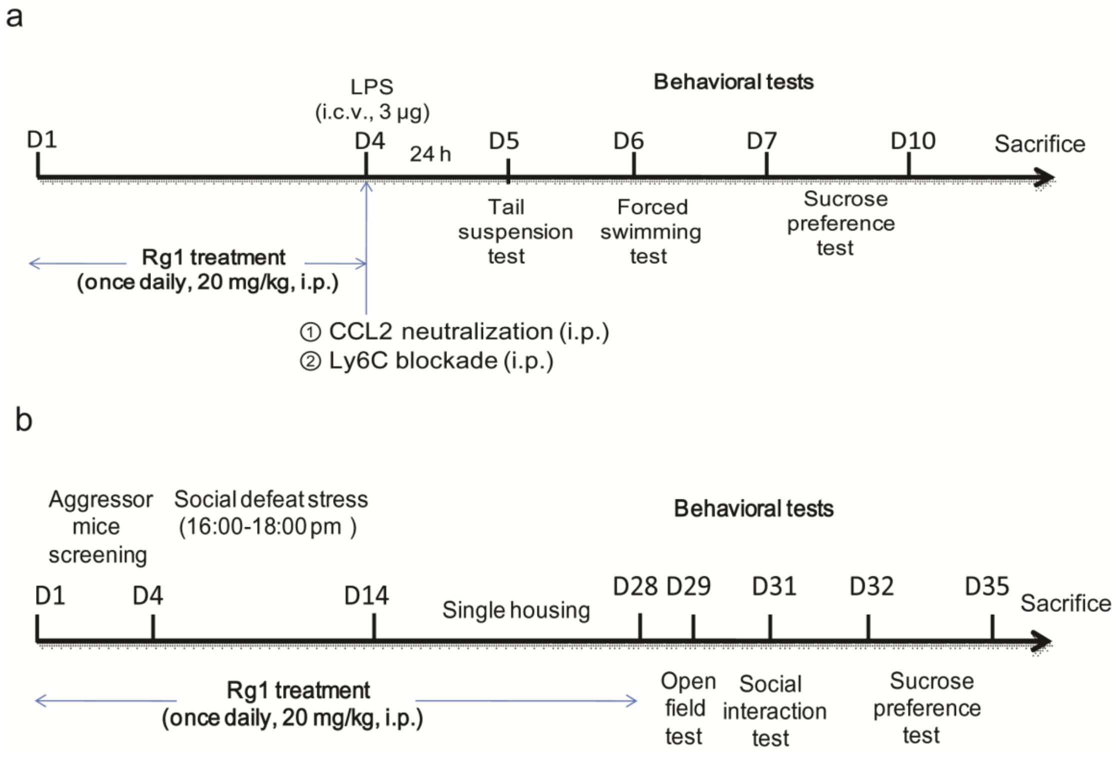

Supplementary Figure S6. **Timeline for drug administration and conduction of behavioral tests.** (**a**) In neuroinflammation-induced depressive mice model, Rg1 was administered once daily for 4 days before the central intracerebroventrical (i.c.v.) LPS challenge. 24 h later, the mice were evaluated for the depression-like behaviors by tail suspension test, forced swimming test and sucrose preference test in the following 5 days. (**b**) Mice were subjected to a 10-day social defeat stress paradigm to induce depression-like behaviors. Rg1 was administered once daily for 28 days before the initiation of a battery of behavioral tests to assess the anxiety and depression-like behaviors.

Supplementary Methods

Immunohistochemistry

Mice were perfused transcardially with about 200 mL of 10% PBS-buffered formalin via the ascending aorta. The brains were carefully removed and fixed overnight in 10% formalin plus 20% sucrose before paraffin embedding and coronal sectioning (8 μm). Antigen retrieval was performed with EDTA. After quenching endogenous peroxidase with 0.3% H_2O_2 for 10 min, the sections were blocked by 10% goat serum for 1 h at room temperature. Then, sections were incubated overnight at 4 ℃ with primary anti-GFAP antibody (1:800, Epitomics, USA). After rinsing with PBS for 3 times, the sections were incubated with biotinylated secondary antibody for 30 min at room temperature. Immunoreactivity was visualized by 3, 3-diaminobenzidine (DAB). Sections were washed thoroughly, mounted onto gelatin-coated slides, dehydrated and coverslipped before being examined by microscopy (Leica, Germany). GFAP staining intensity was assessed using Image J software (Bethesda, MD, USA), and the average optical density was used for the statistical analysis of GFAP intensity (3 sections were quantified per mouse brain). A non-stained region was selected and used as the background. All the sections were examined by pathologists in a blinded manner.

Immune cell isolation

To isolate infiltrated immune cells from mice brain, the hemispheres were carefully minced to homogenates with ice-cold PBS. After resuspension with 100% Percoll medium to generate 30% Percoll system, the mixtures (10 mL) were carefully layered onto the 70% Percoll system (2 mL), centrifuged at 500 × g, 18 ℃ for 30 min. The cell layers at the 30/70% interface were carefully collected, thoroughly washed before further analysis.

To isolate the mononuclear cells from blood and spleen homogenates, the lympholyte-Mammal separation medium was utilized. Briefly, blood was diluted 1:1 with PBS (v/v), precisely layered onto the surface of the Lympholyte-M medium and centrifuged at 1200 × g for 20 min under room temperature. Then, the cell layer at the interface was carefully transferred into a 15-ml centrifuge tube and thoroughly washed with PBS. The remaining red blood cells were removed with lysis buffer.

Flow cytometry

The flow cytometric analysis was carried out with standard methods. Briefly, cells were firstly

incubated in anti-CD16/32 blocking solution, and stained at 4 ℃ for 30 min with specific antibody cocktails as follows: anti-CD45-PE, anti-CD45-Percp, anti-Ly6C-FITC, anti-Ly6C-PE, anti-CD11b-APC, anti-Ly6G-Percp, anti-CD4-Percp. After washing, the samples were stained with FITC-conjugated anti-goat secondary antibody for 30 min. After all staining was completed, the samples were washed twice in PBS and fixed in 1% paraformaldehyde before analysis. Data acquisition was performed on a FACSCalibur flow cytometer (BD Biosciences, USA) and analyzed with the FlowJo software (TreeStar).

Quantitative real-time PCR

Total RNA was isolated from the brain and cell samples using 1 mL of Trizol reagent (Takara, Japan). Total RNA (0.5 μg) was reverse-transcribed to cDNA using a PrimeScript RT Master Mix kit (Takara, Japan). Real-time qPCR reactions were carried out in a total volume of 15 μM with SYBR Green I PCR mix kit (Takara, Japan). The mRNA concentrations of all detected genes were normalized to that of β-actin. The determination of the mRNA fold changes was carried out using the $2^{-\Delta\Delta CT}$ method. Detailed primer sequences for the targeted genes are listed as follows:

Target genes	Forward (5'-3')	Reverse (5'-3')
IL-1β	CTGTGTCTTTCCCGTGGACC	CAGCTCATATGGGTCCGACA
TNF-α	TTCTGTCTACTGAACTTC	CCATAGAACTGATGAGAG
IL-6	CCAGAAACCGCTATGAAGTTCCT	CACCAGCATCAGTCCCAAGA
iNOS	ATTTGGGAATGGAGACTGT	TGAAGGTGTGGTTGAGTT
CCL2	AGATCAGAACCTACAACT	GGTCAACTTCACATTCAA
CCL3	CAATTCATCGTTGACTATT	GGTCAGTGATGTATTCTT
CCR2	CTATCAACATCTCATTCTCTATT	CACCATCATCGTAGTCAT
β-actin	TCTGGCACCACACCTTCTA	AGGCATACAGGGACAGCAC

Western blotting analysis

The protein samples were separated on a 10% SDS-polyacrylamide gel and transferred onto a polyvinylidene difluoride membrane (Bio-Rad, CA, USA). The membrane was blocked with 5% bovine serum albumin or defatted milk, followed by incubation with rabbit monoclonal anti-mouse phospho-Akt (Ser-473), Akt, phospho-p38 MAPK (Thr-180/Tyr-182), p38 MAPK, phosphor-Erk and Erk primary antibodies overnight at 4 ℃ (Cell Signaling Technology, USA). Following the incubation with a horseradish peroxidase (HRP)-conjugated secondary antibody, the signals were detected using an enhanced chemiluminescence kit (Thermo Fisher Scientific,

Waltham, MA, USA) and were captured using a ChemiDoc XRS+ System (Bio-Rad, CA, USA).

LC-MS/MS determination of neurotransmitter and their metabolites

Briefly, the brain cortex (50 mg) was homogenated in ice-cold water (containing 20 mM ascorbic acid and 5.0 μg/mL caffeic acid) and the protein was precipitated with ice-cold acetonitrile (v:v=1:4). The supernatant (500 μL) was then evaporated to dryness under vacuum. For derivatization, 25 μL of borate buffer (sodium tetraborate, 100 mM in water) and 25 μL of benzoyl chloride (2.0% in acetonitrile, v:v) were added to the residue and vortexed under room temperature for 5 min. After centrifugation at 18,000 rpm for 10 min, 30 μL aliquot was transferred to the vial and 5 μl was injected for analysis. In the LC-MS/MS analysis, the chromatographic separation was achieved on a Kromasil C_{18} 2.1 mm × 150 mm column (Akzo Nobel, Bohus, Sweden) with the column temperature set at 40 ℃. The mobile phase consisted of solvent A (0.1% formic acid and 2.0 mM ammonium acetate in water) and solvent B (acetonitrile). The mobile phases were eluted at 0.2 mL/min following the gradient as follows: 30% B maintained for 1.5 min, increased to 65% at 3.5 min and held for 1.5 min, increased to 75% at 8.0 min, then decreased to 30% at 10.0 min followed by 3.0 min for equilibration. The flow was diverted to the waste in the initial 4.5 minutes. The mass spectrometer was operating at the following parameters: ionspray voltage, 5.0 kV; source temperature, 550 ℃; curtain gas, 20; CAD gas, 9; nebulizer gas (GS1), 55; auxilliary gas (GS2), 50. The dwell time was set at 30 ms for each ion transition. The electrospray ionization (ESI) source was operated in the positive mode. MRM monitoring conditions for dopamine, serotonin, 5-hydroxyindoleacetic acid, kynurenic acid and 3-hydroxykynurenine were 466.0-241.2, 385.0-264.2, 313.0-146.0, 433.0-294.0, 293.9-105.1, respectively.
